# “Small” Large Language Models in the Hospital: Evaluation Study on Real-World Data in a Resource-Constrained Setting

**DOI:** 10.2196/86453

**Published:** 2026-09-09

**Authors:** He A Xu, Romain Pythoud, Christian W Thorball, Giorgia Carra, Bogdan Kulynych, Jérémie Despraz, Coralie Galland-Decker, Errikos Maslias, Edouard Baudson, Thomas Brahier, Vanessa Kraege, Ana Catarina de Sousa Teixeira, Carlos Fidalgo, Florian Berthaudin, Amagoia Madina, Solange Zoergiebel, Francois Bastardot, Athina Stravodimou, Marie Méan, Jacques Fellay, Philippe Ryvlin, Jean Louis Raisaro

**Affiliations:** 1 Biomedical Data Science Center University Hospital of Lausanne Lausanne Switzerland; 2 Infectious Diseases Service University Hospital of Lausanne Lausanne Switzerland; 3 Clinical Informatics Unit University Hospital of Lausanne Lausanne Switzerland; 4 Department of Clinical Neurosciences University Hospital of Lausanne Lausanne Switzerland; 5 Lausanne University hospital Innovation and medical research directorate Lausanne Switzerland; 6 University of Lausanne Faculty of Biology and Medicine Lausanne Switzerland; 7 Lausanne University Hospital and University of Lausanne Division of Internal Medicine Lausanne Switzerland; 8 IT Department University Hospital of Lausanne Lausanne Switzerland; 9 Department of Oncology, Medical Oncology Service University Hospital of Lausanne Lausanne Switzerland; 10 School of Life Sciences École Polytechnique Fédérale de Lausanne Lausanne Switzerland

**Keywords:** clinical NLP, evaluation framework, French medical text, health care, hospital deployment, large language model, LLM, natural language processing, resource-constrained settings, small language models

## Abstract

**Background:**

Large language models (LLMs) are increasingly being deployed in health care, but their use and deployment in many real-world hospital environments pose significant challenges and concerns. In particular, state-of-the-art commercial models store or process data externally, which is often in conflict with ensuring patient data protection. At the same time, using LLMs locally is limited by the lack of available computing infrastructure. Small open-source LLMs that do not require substantial computing resources could offer a practical way to resolve these tensions, but their medical utility in real-world local contexts, especially in non-English languages, has not been sufficiently evaluated.

**Objective:**

This study aimed to evaluate the feasibility of small, locally deployable open-source LLMs for clinically relevant tasks in a resource-constrained hospital setting and to propose a reproducible framework for institution-specific evaluation before deployment.

**Methods:**

We evaluated 6 open-source LLMs ranging from 8B to 24B parameters (from the Mistral, Phi4, Falcon3, Llama3.1, and Meditron3 families) in a zero-shot setting across 7 tasks covering 4 clinical use cases: information extraction, medical text translation, text generation, and clinical decision support. We used deidentified French clinical data from a Swiss tertiary hospital, including discharge letters, clinical notes, and structured electronic health records. Performance was assessed using task-specific metrics, such as precision, recall, *F*_1_-score, embedding-based semantic similarity, recall-oriented understudy for gisting evaluation (ROUGE) score, readability indices, and human review by clinicians.

**Results:**

Model performance varied substantially between tasks. In the simplest retrieval task (needle-in-the-haystack), several models performed strongly, with Llama3.1 achieving an *F*_1_-score of 99.81% and Mistral-small achieving 99.71%. In contrast, performance was poor in more complex tasks. For detecting protected health information, the best-performing LLMs achieved only modest overall macro–*F*_1_-scores (0.33-0.34), substantially below a fine-tuned Robustly Optimized BERT Pretraining Approach (RoBERTa) baseline (0.94). In the task of extracting immune-related adverse events from discharge notes, the highest overall macro–*F*_1_-score was 0.35 with Phi4. For medical text translation, Phi4 ranked highest in embedding-based evaluation, whereas Meditron3-Phi4 performed the worst, with clinician reviews identifying hallucinations in 55% of its outputs. In the task of summarizing discharge letters, quality was low across all models, with the best penalized ROUGE score reaching only 0.169 with Llama3.1. In the tasks of generating patient-friendly discharge note summaries and clinical decision support, clinician ratings generally ranged from dissatisfied to neutral, and no model achieved consistently satisfactory performance.

**Conclusions:**

Small open-source LLMs appear feasible for simple retrieval-oriented tasks in local hospital deployments but are currently inadequate for more complex applications, such as clinical decision support, deidentification, extraction of adverse events, and medical summarization. These findings highlight the importance of locally grounded evaluation tailored to specific use cases and the need for robust institutional evaluation frameworks to ensure safe and reliable deployment.

## Introduction

Large language models (LLMs) attain high performance in medical tasks such as exam-style question answering according to benchmark-based evaluations [1,2]. As opposed to classical statistical or machine learning systems, which often require structured data, LLMs work directly with text, the predominant data representation in medicine and a convenient free-form interface for practitioners to interact with models [3]. This versatility and the observed high performance in standardized benchmarks prompted advocates across academia [4] and industry [5] to call for broader adoption of LLMs into medical informatics systems, for example, for decision support or assistance in operational and administrative tasks [6].

Despite this recent push, the adoption of LLMs in hospital environments poses significant challenges and concerns. First, the utility of many current LLMs is constrained by the data on which they are trained and tested. A significant portion of medically specialized LLMs is pretrained or fine-tuned using publicly available datasets such as PubMed and tested on variations of MultiMedQA [1] multiple-choice datasets. These data do not fully represent the complexity and heterogeneity of real-world clinical use cases and the nuances of clinical notes, limiting their generalizability and performance [7]. Second, although many LLMs are pretrained on multilingual corpora, their performance in non-English clinical contexts remains insufficiently studied. In addition, the practical integration of LLMs into existing hospital information systems poses substantial technical, logistical, and ethical issues due to data protection mandates, high computational requirements, legacy interfaces and infrastructures, and the need to control the model life cycle [8], all of which require careful consideration for safe and successful deployment [9]. Moreover, the regulatory frameworks differ significantly across the world. For instance, the United States primarily regulates health AI through sectoral rules such as the Health Insurance Portability and Accountability Act (HIPAA) [10] and US Food and Drug Administration (FDA) guidance, whereas in the European Union (EU), the primary regulatory frameworks are the General Data Protection Regulation (GDPR) and the forthcoming EU AI Act, both of which impose strict requirements on data processing, transparency, and model accountability. The regulations significantly restrict the sharing of health data with external cloud services and strongly motivate on-premises or locally hosted deployments to ensure compliance and maintain institutional control over patient information.

Existing research has largely focused on evaluating LLMs (often exceeding 70 billion parameters) available through APIs as a service across various health care applications and on simulated or publicly available datasets [11,12]. Yet, most health care institutions do not have the computational resources to locally host any of these models and cannot transfer sensitive patient data to the cloud due to ethical commitments, data protection regulations, and internal policies. In contrast, smaller LLMs (typically under 24 billion parameters), which are more suitable for resource-constrained settings and practical deployments, have received comparatively little attention.

To address this gap, this study introduces a reproducible evaluation framework for assessing smaller, computationally efficient LLMs on institution-specific data. The objective is to go beyond standardized and open medical evaluation benchmarks that often fail to capture local specificities and language. We demonstrate the utility of this framework in 2 ways. First, we characterize the performance of these models under realistic operational conditions by evaluating a suite of smaller LLMs on authentic French-language clinical tasks within our hospital, a tertiary university hospital in Switzerland. Second, we provide a methodological template that enables researchers and other health care institutions to validate and select appropriate models for local deployment, particularly in environments with limited computational resources. This work ultimately investigates the viability of accessible LLMs to support clinical practice in local contexts.

## Methods

### Ethical Considerations

The Legal Affairs Unit of Lausanne University Hospital confirmed that this study falls outside the scope of Swiss research legislation (Federal Act on Research involving Human Beings, Human Research Act, HRA, SR 810.30). Consequently, formal authorization from an ethics committee was not required. However, in accordance with institutional and legal standards, the study was conducted exclusively using data for which informed consent had been previously obtained.

### Overview

In this section, we evaluated 4 representative use cases—information extraction, medical text translation, text generation, and clinical decision support—to reflect common scenarios encountered in hospital environments. Multiple language models were selected and assessed across these tasks to compare their performance.

### LLM Selection and Configuration

Model selection was guided by institutional computational capacity and regulatory restrictions preventing the use of external APIs or cloud services for patient data. Accordingly, we limited our evaluation to open-source LLMs with 1 to 24 billion parameters and excluded larger models (eg, 70B), even in quantized form, to avoid confounding performance effects.

Current approaches to leveraging open-source LLMs in health care typically involve two main strategies: (1) fine-tuning pretrained models on specific medical data to enhance their performance on specialized tasks or (2) improving either general-purpose or medically fine-tuned LLMs directly through prompt engineering or incorporating external data as part of prompts to guide the outputs for clinical relevance [13]. We focused only on pretrained models because fine-tuning requires additional computational resources, which are usually not available in many health care institutions. Additionally, we selected models that were state of the art at the time of analysis.

Given the model size constraint, we initially considered a diverse set of models within the 1 to 24 billion parameter range. A critical selection criterion was sufficient pretraining on French-language corpora, as the clinical data in our setting were predominantly in French. Furthermore, we required models to support a minimum context window of 8000 tokens to adequately process the typical length of medical documents encountered in our hospital. Models that did not meet these requirements were excluded. For instance, models such as BioGPT [14] and BioMistral-7B [15] were not considered due to their limited context window, while others (eg, MedLM [16]) were excluded due to the lack of publicly available model weights, preventing reproducible evaluation. Similarly, BioMedGPT-LM-7B [17] was excluded because, despite its domain-specific fine-tuning, it lacked instruction tuning necessary for the intended applications. Based on these criteria, we selected the following 6 open-source models for evaluation: Mistral-Small-24B-Instruct-2501 [18], Phi4-14B [19], Falcon3-10B-Instruct [20], Llama3.1-8B-Instruct [21], and Meditron3-Phi4-14B and Meditron3-8B-Instruct [22]. While we acknowledge that several other high-performing models exist, limitations related to context window size and/or accessibility of model weights precluded their inclusion; future work may extend this evaluation to such models as they become available.

To maintain consistency and assess the inherent capabilities of these smaller language models without the influence of few-shot examples—which can be challenging to optimize within limited context windows and may introduce variability—this study exclusively evaluated the LLMs in a zero-shot setting. This approach involves providing instructions directly to the model without any preceding examples or the help of external tools such as web search or retrieval-augmented generation (RAG) techniques [23].

All experiments were conducted on an internal server using the Hugging Face Transformers library (version 4.36.2). Hardware resources included 3 GPUs: 2 NVIDIA Quadro M6000 and 1 Quadro P6000, each with 24 GB of VRAM (72 GB VRAM in total). We evaluated dense transformer architectures without quantization. The pretrained model weights were obtained from the Hugging Face platform [24]. For models natively distributed in Brain Floating Point 16-bit (BF16) precision, as our GPUs do not support this precision, the model weights were upcast to full 32-bit floating-point precision (FP32) during loading. This upcasting increased the memory footprint of the model weights. To facilitate inference within our 72 GB VRAM capacity, we used a hybrid offloading strategy using the *device_map='auto'* configuration. This sharded parts of the model layers across the 3 GPUs, while the remaining layers were offloaded to system RAM.

Generation parameters were fixed for all use cases, employing greedy decoding with a maximum output sequence length of 8000 tokens. For reproducibility, prompt templates and system instructions are provided in Multimedia Appendix 1.

### Data Acquisition and Preparation

We conducted the study using deidentified patient data extracted from the clinical data warehouse of our hospital. We included only data from patients who had provided general informed consent for the secondary use of their clinical data for research purposes. The exact data used to test the use cases depended on the use case. Details for each use case are described in the following sections.

We performed all data processing and analyses locally. To ensure patient confidentiality and comply with data protection regulations, all protected health information (PHI) was deidentified prior to its use in this research except for the named entity recognition (NER) use case (a subtask of the information extraction use case). We used a customized text deidentification pipeline previously developed and validated by a subset of the authors, as described in another study [25]. To preserve the temporal integrity of medical information, all dates within each clinical document were shifted by the same randomly selected offset (between 1 and 3 years), thereby preserving relative temporal relationships and clinical timelines for clinically relevant use cases.

**Figure 1:**
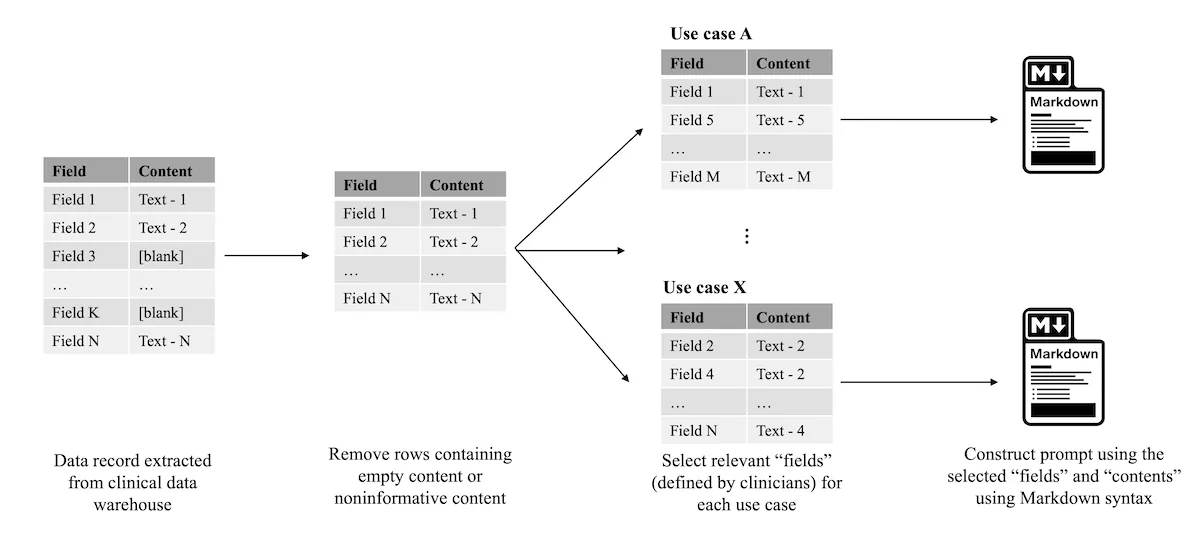
Data extraction and preparation for use cases. Field (short text describing what type of information is presented in “content”) formats in the raw data were automatically detected, and empty or noninformative fields were removed. The remaining content was reordered based on clinician-defined preferences or task requirements, and irrelevant information was filtered out. Finally, the processed data were structured into standardized Markdown prompts, with titles and headers reworded to optimize clarity and relevance for the language models.

To ensure the generation of clean, well-structured prompts for model input, we implemented a comprehensive data preprocessing pipeline. This process involved several key steps, as described in Figure 1. We presented all prompts in French and prompted the models to respond in French to ensure consistency with the language of the discharge letters, except where otherwise specified in the use-case descriptions.

### Use Case Selection and Design

#### Information Extraction

##### Needle-in-the-Haystack

To assess the models’ capacity to locate specific information embedded within clinical documents, we implemented a needle-in-the-haystack evaluation designed to measure token-level information extraction rather than complex medical reasoning. The “needle” consisted of 2 interdependent fabricated sentences that were highly unlikely to occur naturally within our data: “The dinosaur is called Jackson” (“Le dinosaure s'appelle Jackson”) and “Jackson is purple” (“Jackson est pourpre”). These sentences, which collectively provide the answer to the question “What color is the dinosaur?” (“Quelle est la couleur du dinosaure?”), were strategically inserted at distinct points (the first and third quartiles) within each “haystack” document. In our implementation, both the needle and the question were in French. The fictional nature of these sentences ensured no semantic overlap with the original medical content, and their structure allowed for unambiguous answer verification, for example, through keyword matching. Responses provided in either English or French conveying the correct color (eg, “purple” or “pourpre”) were considered correct. We chose this approach to evaluate both the direct retrieval capabilities of the LLMs and their ability to connect related pieces of information separated by substantial intervening text.

The haystack documents consisted of a dataset of 500 French medical discharge letters selected randomly from our data warehouse. We injected the needles into half of the documents and left the other half without needles. Each letter contained between 1000 and 3000 words and featured complex medical language, thereby constituting a challenging and clinically relevant test bed for LLM performance.

##### NER

Building on the assessment of information extraction, the subsequent task focused on NER to specifically extract PHI in clinical notes. We targeted 8 distinct PHI entities: “Name,” “Age,” “Address,” “Telephone,” “Date,” “Time,” and “ID,” selected from a subset of the HIPAA guidance. This evaluation aimed to assess the LLMs’ effectiveness in identifying sensitive personal data embedded within clinical narratives, a foundational capability for developing automated deidentification pipelines to limit privacy risks when sharing medical documentation across institutions or departments. We used an annotated NER dataset of 3687 records, including discharge letters, consultation letters, laboratory reports, and other clinical documents [25], to test the performance of the LLMs.

##### Immune-Related Adverse Event Detection

To assess the LLMs’ performance in a realistic clinical context, the third task focused on the extraction of immune-related adverse events (irAEs) from discharge notes originating from our hospital’s oncology department. The targeted irAEs comprised colitis, thyroiditis, pneumonitis, skin-related effects, hepatitis, hypophysitis, rheumatological manifestations, cardiac toxicities, neurological complications, pancreatitis, nephrological issues, and diabetes. This assessment aimed to test the LLMs’ ability to accurately identify and retrieve specific, clinically significant information from complex medical narratives, mirroring a common requirement in routine oncological practice. We used an annotated irAE dataset of 400 discharge letters to test the performance of the LLMs.

#### Medical Text Translation

As demographics shift toward greater linguistic diversity, the translation of discharge notes into patients’ native languages has become a clinical and administrative imperative. This practice is necessary not only to bridge communication gaps and maintain continuity of care but also to ensure institutional compliance with legal mandates and accurate billing procedures [26]. However, the automated translation of these documents presents a significant challenge. This difficulty is primarily attributed to the requirement for precise and contextually accurate translation of specialized medical terminology.

In this use case, we focused on the models’ ability to accurately translate clinical discharge letters from French to English using a randomly selected dataset of 20 discharge letters collected from different clinical services.

The choice of English as the target language was motivated by its status as a high-resource language in the training of LLMs and its importance for international clinical communication and research dissemination. Successful performance in this task therefore reflects not only translation capability but also accurate comprehension of specialized medical terminology. The prompt instructions were provided in English, and models were required to generate outputs in English (Multimedia Appendix 1). This choice was motivated by technical considerations: most instruction-tuned LLMs are predominantly optimized for English instruction following, leading to more stable and reproducible behavior when prompts are issued in English [27,28]. Using English prompts therefore allowed us to standardize evaluation conditions and reduce variability due to instruction misinterpretation. At the same time, this setup reflects a realistic clinical scenario in which non-English medical documents are translated into English for broader use. We acknowledge that this cross-lingual prompting strategy may introduce confounding effects, which are further discussed in the Limitations section.

#### Text Generation

##### Overview

We assessed the clinical utility of LLMs using 2 text generation tasks targeting different end users. The first task assessed the generation of concise summaries of discharge letters intended for health care professionals. The second task evaluated the models’ ability to translate complex medical information into simplified, patient-friendly language.

##### Generate Discharge Letter Summaries

The first task tested how well the LLMs could summarize key events, diagnoses, and treatments from patient health records, reflecting the real-world challenge clinicians face in managing vast amounts of patient information and medical research. For this evaluation, we randomly selected 40 discharge letters from diverse clinical services and prompted the LLMs to generate a summary of approximately 300 words.

##### Generate Patient-Friendly Discharge Notes

The second task assessed the ability of LLMs to generate patient-friendly discharge notes. The main goal was to translate key clinical information—including diagnoses, treatments, and follow-up instructions—into language understandable to patients with diverse educational levels. There is evidence that this functionality can enhance patient-clinician communication, patient engagement, and continuity of care. For the experiment, we randomly selected 14 deidentified discharge letters from the internal medicine department. Although the prompt was designed in English for multilingual use, the model outputs were generated in French and were subsequently evaluated for clinical accuracy and clarity by 2 native French-speaking clinicians.

#### Clinical Decision Support

This use case assessed the LLMs’ capabilities in clinical decision support, specifically their ability to generate differential diagnoses and propose treatment plans. We consider this task to be one of the most challenging assessments of LLM performance in the medical domain.

For this task, we intentionally excluded discharge letters and instead provided the LLMs with curated prompts containing comprehensive patient information, including anamnesis, consultation notes, laboratory results, vital signs, and results of clinical examinations. This design aimed to simulate a real-world clinical decision-making scenario in which the diagnosis had not yet been formally established or documented. The objective was to evaluate the models’ ability to integrate heterogeneous clinical data to generate plausible differential diagnoses and suggest appropriate management strategies. We randomly selected 10 deidentified medical cases from the neurology service for this task.

### Evaluation Methods

#### Overview

We evaluated model performance according to the specific nature of each use case, using metrics appropriate to the task. For tasks for which ground truth was available, such as information extraction or translation, we applied quantitative measures (eg, *F*_1_-score and recall-oriented understudy for gisting evaluation [ROUGE] score [29]). For tasks with more subjective or generative outputs, such as text generation or clinical decision support, we assessed performance through structured expert review. This approach ensures that comparisons reflect both task-specific accuracy and practical utility in a clinical context.

#### Evaluation of Information Extraction

We measured the performance of the LLMs on the information extraction tasks using ground truth data.

For the needle-in-the-haystack subtask, we calculated recall, precision, and the *F*_1_-score. Recall reflects the proportion of correct answers regarding the needle (dinosaur’s color). Precision reflects the proportion of retrieved answers that were correct, indicating the reliability of the model’s responses. The *F*_1_-score, defined as the harmonic mean of precision and recall, provides a single summary measure that balances detection sensitivity and prediction accuracy.

In the NER subtask for PHI, we assessed performance using precision, recall, and the macro-average *F*_1_-score. We computed these metrics for each targeted PHI category by comparing LLM-extracted entities against a manually annotated ground truth dataset.

Similarly, for the irAE extraction subtask, we compared the LLM outputs against expert-annotated immune-related adverse events in each discharge letter. We calculated precision, recall, and the macro-average *F*_1_-score for each predefined irAE type to evaluate extraction performance.

To evaluate the extraction performance of the LLMs, we used a previously fine-tuned Robustly Optimized BERT Pretraining Approach (RoBERTa)–based NER model as a baseline. The model was trained on 3010 annotated French clinical documents extracted from the Lausanne University Hospital data warehouse [25]. Because the corpus is in French, we selected the CamemBERT model [30] as the base model. The model was fine-tuned for 60 epochs using a learning rate of 2e–5 and a weight decay of 0.001. We used a consistent batch size of 32 for both training and evaluation phases. To optimize model selection, performance was evaluated at the end of each epoch, and the best-performing weights were automatically restored at the conclusion of the training process to ensure maximum generalizability.

#### Evaluation of Medical Text Translation

We used 2 approaches to evaluate the quality of medical text translation.

First, we used an automated and scalable assessment of translation quality using an LLM-based embedding approach. We encoded both the original French source texts and their LLM-generated English translations into vector representations within a shared multilingual embedding space. We then quantified the semantic similarity between a source text and its translation by calculating the cosine similarity score between their respective vector embeddings. This approach is predicated on the principle that texts conveying the same meaning should exhibit high semantic overlap and thus be close in the embedding space, even when expressed in different languages [31].

For a robust analysis, we selected several distinct embedding models. The criteria for their inclusion were: (1) the model should have independent training processes to reduce shared biases, (2) it should be trained on multilingual data that included both French and English, (3) it should have a context window capacity of at least 8192 tokens to handle potentially long medical documents, and (4) it should have demonstrated strong overall performance on established embedding benchmarks. Thus, we chose the following models for this evaluation: gte-multilingual-base [32], bge-m3 [33], and nomic-embed-text-v1.5 [34].

We also conducted human evaluations by inviting 4 clinicians to evaluate 20 discharge letters randomly selected from various clinical services. They evaluated the quality of the translated text against the original French text using a 5-point Likert scale (1=very dissatisfied; 5=very satisfied). These criteria included:

- Accuracy in translation: is the overall meaning preserved well?
- Completeness: are there any facts missing?
- Readability: does the translation sound natural and easy to read?
- Terminology: is the specialized vocabulary (especially medical terms) translated correctly?
- Safety in translation: does the translation contain any harmful mistranslation that may not be safe for patients? If there is harmful information, the translation is not considered safe.
- Structural quality: is the structure of the letter correctly preserved? Is there any misplaced information?

We also asked the clinicians to indicate whether there were any hallucinations in the translated text (ie, whether the translation contained information that did not exist in the original letter). Clinicians assigned a score of 0 (no hallucination) or 1 (with hallucinations).

To assess the consistency of rater scores across models and evaluation criteria, we computed a distribution-based agreement index for each model-criterion combination. Because the 4 clinicians evaluated nonoverlapping subsets of selected letters, direct pairwise comparison of individual scores was not possible. Instead, for each cell defined by a model m and criterion c, we first computed each clinician’s mean score across their assigned letters, yielding one aggregate score per clinician.

As the original ratings were provided on a 5-point Likert scale (range 1-5), we linearly normalized them to the (0,1) interval prior to the analysis. We then quantified the dispersion of these rater-level means using the SD. Agreement was defined as:

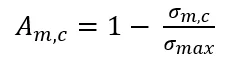



where σ*_m,c_* is the SD of the 4 clinicians’ normalized mean scores for model m and criterion c. Further, σ*_max_* (0.5) is the theoretical maximum SD for a bounded scale in the interval (0,1). This normalization maps agreement to a value between 0 and 1. A value of 0 indicates maximal disagreement, whereas a value of 1 indicates perfect consensus.

#### Evaluation of Text Generation

Evaluating the quality of LLM-generated medical summaries for clinicians presents distinct challenges. Although human assessment by medical professionals would offer the most direct and clinically relevant measure of an LLM’s summarization capabilities, aligning with their specific preferences and informational priorities, this approach was infeasible for the current study due to its time-intensive nature and the limited availability of expert evaluators. Consequently, we used automated benchmarking metrics. The cosine similarity approach, effective for the prior translation task, is less suitable for summarization due to several key limitations. First, significant structural differences exist between lengthy source documents and concise summaries (which often omit details such as extensive laboratory tables and may use formats such as bullet points). Second, maintaining semantic integrity in medical text requires the exact retention of domain-specific terms. Paraphrasing critical medical concepts can introduce ambiguity or error, which may not be reflected by embedding-based similarity measures. Finally, cosine similarity lacks a mechanism to penalize excessive length or direct copying. To address these issues, we used the penalized ROUGE score [29] (equations 2-4) as a more suitable metric for evaluating these summaries.

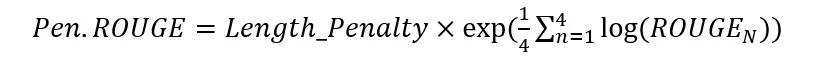

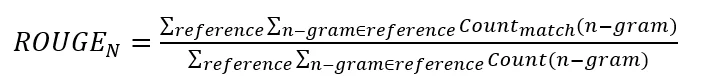

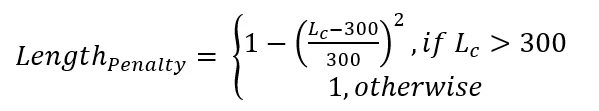







where *Count_match_*(*n*-*gram*) represents the number of n-grams co-occurring in both the generated and reference texts, and *Count(n-gram)* is the total number of n-grams in the reference text. ROUGE-N scores range from 0 to 1, with higher values indicating better recall of key information. *L_c_* represents the number of words in the generated text.

Evaluation of patient-friendly discharge notes combined automated readability assessment with in-depth manual clinical review. We first applied an automated readability metric, the Kandel-Moles index [35], to evaluate readability in French across a diverse set of discharge letters selected to cover various departments and specialties. This index is roughly analogous to the Flesch Reading Ease score for English. Then, the clinicians conducted manual assessments to verify the retention of key clinical facts and overall quality. This was conducted using a structured evaluation grid with 11 criteria, each rated on a 5-point Likert scale (1=very dissatisfied; 5=very satisfied). These criteria included:

- Accuracy (diagnosis, treatment plan, patient instructions, and avoidance of distortions or hallucinations)
- Completeness (no omitted information)
- Safety (no clinically significant errors)
- Readability (ease of reading and minimal jargon)
- Tone (appropriate for patients)
- Logical structure (clear information hierarchy)
- Anonymization (no personal identifiers)
- Ethics (no biases)
- Overall report quality.

Two clinicians independently evaluated 6 model-generated responses for each of 14 discharge letters to ensure a robust assessment.

#### Evaluation of Clinical Decision Support

Our initial attempts to automate the evaluation of LLM-generated clinical decision support proved ineffective. Standard automated metrics were unsuitable for 2 principal reasons. First, there was a structural mismatch. The LLM outputs, generated in a zero-shot setting, did not conform to the structure or format of clinician-authored notes. This rendered metrics based on semantic vector similarity, such as cosine similarity, unreliable for comparing the model’s response to the ground truth text. Second, there was a lexical discrepancy. Reference clinician reports were typically highly concise and contained numerous hospital-specific abbreviations and medical shorthand. Consequently, string-matching algorithms and metrics based on n-grams (eg, ROUGE) failed to capture semantic equivalence due to low lexical overlap. These challenges necessitated the development of a robust manual evaluation framework leveraging clinical expertise.

We thus designed a manual evaluation protocol in collaboration with practicing clinicians. We recruited a panel of 3 board-certified clinicians to assess model outputs independently. They used a dedicated annotation platform to independently score the responses from 6 different LLMs across 10 unique clinical case prompts. Clinicians rated each response against 7 predefined criteria using a 5-point Likert scale (1=very dissatisfied; 5=very satisfied). The evaluation criteria were:

- Diagnostic and treatment accuracy: correctness of the proposed diagnoses and treatment plans.
- Diagnostic and treatment safety: absence of suggestions that could lead to patient harm.
- Relevance of suggested additional examinations: clinical appropriateness of recommended diagnostic tests to confirm or refute differential diagnoses.
- Logical reasoning: coherence and logical consistency of the clinical reasoning presented.
- Structural quality: clarity, organization, and readability of the response.

#### Statistical Test

To test differences in model performance for the clinical decision support and patient-friendly discharge note generation use cases, we used the Kruskal-Wallis H test for each of the evaluation criteria defined above. Following significant Kruskal-Wallis test results, post hoc pairwise comparisons were performed using the Mann-Whitney *U* test. To control for type I errors across multiple comparisons, *P* values were adjusted using the Bonferroni correction. CIs were estimated using the bootstrap method with 1000 resamples.

## Results

### Overview

For conciseness, in the following text we sometimes refer to the Mistral-Small-24B-Instruct-2501 model as “Mistral-small,” the Phi4-14B model as “Phi4,” the Llama3.1-8B-Instruct model as “Llama3.1,” the Falcon3-10B-Instruct model as “Falcon3,” the Meditron3-8B-Instruct model as “Meditron3,” and the Meditron3-Phi4-14B model as “Meditron3-Phi4.”

### Information Extraction

We evaluated the 6 selected models across 3 information extraction tasks. The detailed performance metrics are presented in Tables 1-3, respectively. In the needle-in-the-haystack task, Llama3.1 achieved the highest retrieval performance (*F*_1_-score=99.81%), with Mistral-small demonstrating comparable results. In contrast, Meditron3-Phi4 showed the weakest performance, exhibiting the lowest recall (61.35%) and an *F*_1_-score of 76.15%.

**Table 1:** Performance of the selected models on the needle-in-the-haystack task, measured by extraction recall, precision, F1-score, and 95% CI.

Model	Size	Recall, %	Precision, %	*F*_1_-score (95% CI), %
Mistral-small	24B	99.42	100	99.71 (98.34-100)
Phi4	14B	86.54	100	92.78 (88.23-96.13)
Meditron3-Phi4	14B	61.35	100	76.05 (67.97-82.58)
Falcon3	10B	95.58	100	97.74 (94.88-99.47)
Llama3.1	8B	99.62	100	99.81 (98.34-100)
Meditron3	8B	87.69	89.76	88.71 (82.53-92.38)

**Table 2:** Performance of the selected models on the PHIa detection task, measured by macro-average F1-score with 95% CI. Higher values indicate better performance. The column headings NAME, DATE, TIME, ID, AGE, TEL, and ADDRESS represent the original PHI entity categories.

Model	Size	NAME	DATE	TIME	ID	AGE	TEL	ADDRESS	Overall (95% CI)
Mistral-small	24B	0.84^b^	0.11	0.83	0.26	0.53^b^	0.06	0.08^b^	0.33 (0.32-0.35)
Phi4	14B	0.00	0.77	0.88	0.51	0.53^b^	0.06	0.01	0.34 (0.32-0.36)
Meditron3-Phi4	14B	0.00	0.89	0.90^b^	0.46^b^	0.43	0.06	0.01	0.34 (0.32-0.36)
Falcon3	10B	0.00	0.90^b^	0.87	0.31	0.53	0.06	0.04	0.34 (0.31-0.35)
Llama3.1	8B	0.00	0.49	0.61	0.16	0.26	0.01	0.04	0.20 (0.17-0.22)
Meditron3	8B	0.00	0.00	0.00	0.00	0.00	0.00	0.00	0.00 (0.00-0.00)
Fine-tuned RoBERTa	—	0.95	0.99	1.00	0.81	0.74	1.00	0.79	0.94 (0.91-0.96)
Support, n	—	541	1336	314	25	138	166	571	3091

^a^PHI: protected health information.

^b^Highest performance among the tested large language models for each PHI entity category.

^c^Not applicable.

**Table 3:** Performance of the selected models on immune-related adverse event extraction, measured by macro-average F1-score with 95% CI. Higher values indicate better performance.

Category	Mistral-small	Phi4	Meditron3-Phi4	Falcon3	Llama3.1	Meditron3
Cardiac	0.36	0.33	0.40	0.31	0.00	0.40
Colitis	0.46	0.54	0.53	0.44	0.54	0.45
Diabetes	0.25	0.55	0.17	0.00	0.00	0.00
Hepatitis	0.38	0.36	0.33	0.29	0.18	0.09
Hypophysitis	0.31	0.32	0.31	0.12	0.12	0.17
Nephrological	0.08	0.22	0.35	0.24	0.21	0.29
Neurological	0.08	0.11	0.11	0.03	0.06	0.00
Pancreatitis	0.55	0.67	0.00	0.57	0.67	0.40
Pneumonitis	0.46	0.53	0.43	0.29	0.22	0.00
Rheumatological	0.07	0.09	0.08	0.00	0.00	0.00
Skin	0.21	0.20	0.26	0.18	0.29	0.18
Thyroiditis	0.30	0.34	0.29	0.31	0.24	0.14
Overall (95% CI)	0.29 (0.20-0.35)	0.35 (0.25-0.42)	0.26 (0.19-0.32)	0.23 (0.15-0.28)	0.21 (0.12-0.26)	0.17 (0.08-0.25)

Performance on the PHI detection task varied across models and PHI categories. Mistral-small achieved the best results for NAME, AGE, and ADDRESS detection. Falcon3 performed best for DATE and AGE, while Phi4 was most effective for ID and AGE. Meditron3-Phi4 yielded the highest performance for TIME detection. Despite these differences, no single model demonstrated consistently high performance across all PHI categories.

We further compared LLM performance with that of a fine-tuned RoBERTa baseline trained on a French clinical dataset from our institution [25], providing a reference point for assessing the performance gap between zero-shot small LLMs and a task-specific fine-tuned model.

Similar to the PHI detection task, model performance in the irAE detection task differed substantially across irAE categories. Models from the Meditron family exhibited superior performance in detecting cardiac adverse events, with Meditron3-Phi4 also achieving the best results for nephrological adverse events. The Mistral-small model was most effective for hepatitis detection. For colitis detection, both the Phi4 and Llama3.1 models demonstrated leading performance. The Phi4 model additionally excelled at detecting diabetes and hypophysitis. Overall, no single model demonstrated consistently strong performance across all evaluated irAE categories.

### Medical Text Translation

Because the exact values of cosine similarity scores are model dependent and not directly comparable, we used a relative comparison rather than an absolute one. We evaluated the models based on their performance rankings. Table 4 summarizes these findings, presenting the overall rank and the detailed ranking for each generative model as determined by the various embedding-based similarity scores. Among the models tested, Phi4 demonstrated the highest translation efficacy, whereas Meditron3-Phi4 yielded the poorest results. Our analysis of the model outputs revealed that the performance degradation of Meditron3-Phi4 is attributable to its fine-tuning process, during which the output was truncated to a 1000-token window despite an 8000-token input capacity, which severely compromised its long-context translation capabilities.

**Table 4:** Overall performance ranking of the selected large language models for the medical translation task from French to English, based on individual ranking results from 3 independent embedding models.

Model	Size	Embedding similarity ranking			Overall rank
		gte-multilingual-base	bge-m3	nomic-embed-text-v1.5	
Phi4	14B	1	1	1	1
Falcon3	10B	2	2	3	2
Mistral-small	24B	3	3	4	3
Meditron3	8B	5	4	2	4
Llama3.1	8B	4	5	5	5
Meditron3-Phi4	14B	6	6	6	6

**Figure 2:**
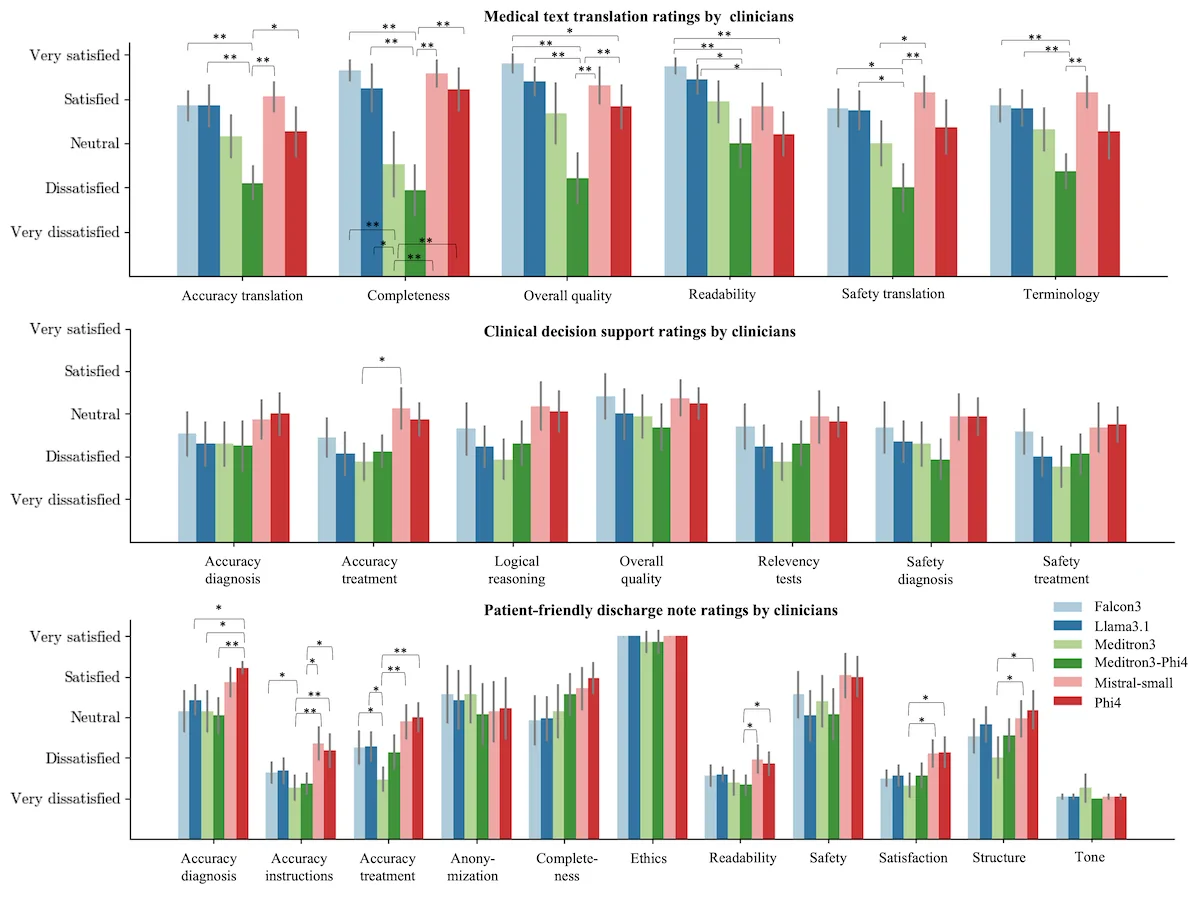
Clinicians’ evaluation of the text generated by large language models (LLMs) in 3 use cases: medical text translation, clinical decision support, and patient-friendly discharge note generation across the predefined evaluation dimensions. Each colored bar represents a tested LLM. Error bars represent the 95% CI. *P<.05; **P<.001. See Multimedia Appendix 2.

The suboptimal performance of Meditron3-Phi4 in the medical translation task was also confirmed by clinician evaluations (Figure 2). This model consistently received “dissatisfied” ratings across all evaluation dimensions, representing statistically significant underperformance compared with the other models. In contrast, Falcon3, Llama3.1, and Mistral-small consistently achieved or exceeded the “satisfied” threshold across all criteria. The Meditron3 and Phi4 models occupied an intermediate tier, with performance ratings aligning with the “neutral” level.

**Figure 3:**
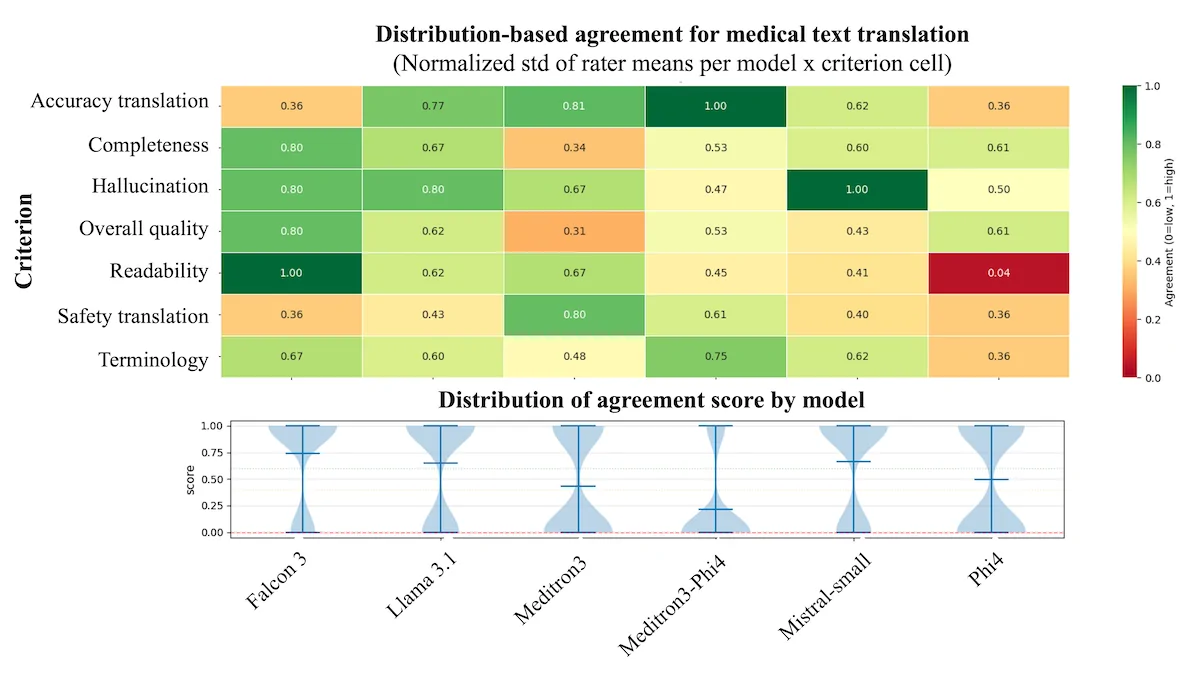
Raters’ agreement for the medical text translation use case. Distribution-based agreement index across models and evaluation criteria. Values range from 0 (maximal disagreement) to 1 (perfect consensus). Higher values indicate greater consistency across clinicians. The violin plots present the distribution of the agreement index across all evaluation criteria for each language model.

Regarding hallucinations, most models demonstrated high fidelity to the source text. As shown in Table 5, Mistral-small achieved a perfect score, with no identified hallucinations (0/20). Llama3.1 and Falcon3 followed closely, each exhibiting hallucinations in only 5% of cases (1/20). In contrast, the Meditron3-Phi4 model emerged as a significant outlier, generating nonexistent information in 55% of the evaluated summaries (mean 0.61, SD 0.32), representing a substantial deficit in clinical factuality compared with its counterparts. Figure 3 presents the distribution-based agreement for each model-criterion combination. Agreement varied across combinations. The readability of the Falcon3 model (rated between “satisfied” and “very satisfied”), the accuracy of translation of the Meditron3-Phi4 model (rated “dissatisfied”), and hallucination ratings for the Mistral-small model (rated “no hallucination”) reached the highest agreement score (score 1, indicating perfect consensus). Clinicians’ agreement on the readability of the Phi4 model was the lowest.

**Table 5:** Evaluation of hallucination rates across the language models (n=20).

Model	Score, mean (SD)	Hallucination count, n/N (%)
Mistral-small	0.00 (0.00)	0/20 (0)
Llama3.1	0.05 (0.22)	1/20 (5)
Falcon3	0.05 (0.23)	1/20 (5)
Phi4	0.11 (0.32)	2/20 (10)
Meditron3	0.11 (0.32)	2/20 (10)
Meditron3-Phi4	0.61 (0.32)	11/20 (55)

### Text Generation

#### Discharge Summary Generation

We detail the performance of selected LLMs on the summarization task, quantified by our proposed penalized ROUGE (Pen ROUGE) metric, in Table 6. The results show that Llama3.1 was the top-performing model, whereas Falcon3 was the least effective. Two key observations emerge from these results. First, the performance gap between the models was small, indicating only minor relative advantages. Second, and more critically, the universally low Pen ROUGE scores (between 0.1 and 0.2) suggest that the overall summarization quality was suboptimal across all tested models, highlighting the inherent difficulty of the task for small LLMs.

**Table 6:** Mean ROUGEa and Pen ROUGEb scores of the selected large language models on the discharge summary generation task, with 95% CIs.

Model	Size	ROUGE↑ (95% CI)	Text length, words (95% CI)	Length penalty (95% CI)	Pen ROUGE↑ (95% CI)
Mistral-small	24B	0.184 (0.14-0.22)	391 (348.98-432.06)	0.833 (0.71-0.95)	0.156 (0.11-0.19)
Phi4	14B	0.151 (0.11-0.19)	368 (345.52-389.53)	0.927 (0.88-0.96)	0.141 (0.09-0.18)
Meditron3-Phi4	14B	0.190 (0.12-0.25)	445 (353.49-536.18)	0.727 (0.54-0.91)	0.114 (0.06-0.16)
Falcon3	10B	0.107 (0.08-0.13)	248 (220.657-276.08)	0.998 (0.99-1.00)	0.107 (0.08-0.13)
Llama3.1	8B	0.180 (0.14-0.22)	316 (275.98-356.53)	0.941 (0.89-0.99)	0.169 (0.13-0.21)
Meditron3	8B	0.287 (0.17- 0.40)	412 (279.78-545.16)	0.730 (0.55-0.90)	0.144 (0.09-0.19)

^a^ROUGE: recall-oriented understudy for gisting evaluation.

^b^Pen ROUGE: penalized ROUGE.

#### Clinical Decision Support

The evaluation of diagnosis and treatment generation revealed suboptimal performance across all models, with none achieving a “satisfied” rating on any evaluation dimension (Figure 2). Most models scored within the “dissatisfied” to “neutral” range. Intermodel statistical analysis showed no significant variation except for accuracy of treatment, for which a significant difference was observed between Mistral-small and Meditron3 (*P*=.03). Although the Meditron models consistently yielded lower scores, the margin of underperformance remained statistically nonsignificant.

**Figure 4:**
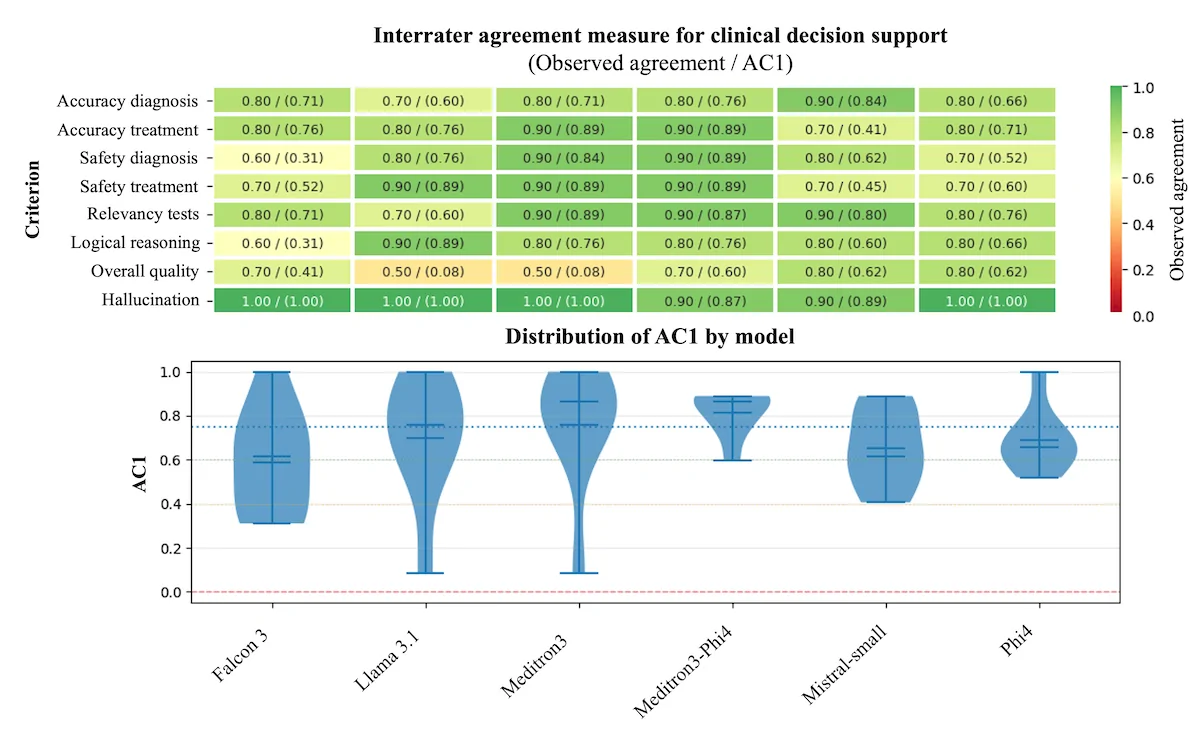
Raters’ agreement for the clinical decision support use case. Interrater agreement was measured by observed agreement and Gwet agreement coefficient, version 1 (AC1). Each column presents the ratings for 1 model; the model name is presented at the bottom of the plot. The color of the heat map represents the observed agreement value. The value in each cell presents both the observed agreement and AC1 value in the format observed agreement/(AC1). The violin plots present the distribution of AC1 values across all evaluation criteria for each language model. Horizontal reference lines indicate standard interpretation thresholds (excellent agreement indicated by the blue dotted line at an AC1 value of 0.75). Wider violins indicate greater variability in AC1 values across evaluation criteria.

Interrater agreement for this use case was evaluated using both observed agreement and Gwet agreement coefficient, version 1 (AC1) [36], to account for potential bias due to skewed rating distributions (Figure 4). Overall, the observed agreement was consistently high across models and criteria, ranging from 0.70 to 0.90, with perfect agreement (1.00) observed for hallucination evaluation in several models. Correspondingly, AC1 values indicated moderate to strong agreement for most task-specific dimensions, including accuracy, safety, and test relevance (AC1 values 0.60-0.89 across models), suggesting robust and consistent annotation for these clinically relevant criteria.

Agreement for logical reasoning and overall quality was more variable, with some models exhibiting lower AC1 values despite moderate observed agreement, reflecting greater subjectivity in these dimensions. Across models, Meditron-based variants generally demonstrated higher interrater consistency, while other models showed more variability depending on the evaluation criterion.

Overall, the concordance between observed agreement and AC1 in this use case suggests that interrater reliability was generally robust, with less pronounced effects of skewed rating distributions compared with other evaluation settings.

#### Patient-Friendly Discharge Notes

**Figure 5:**
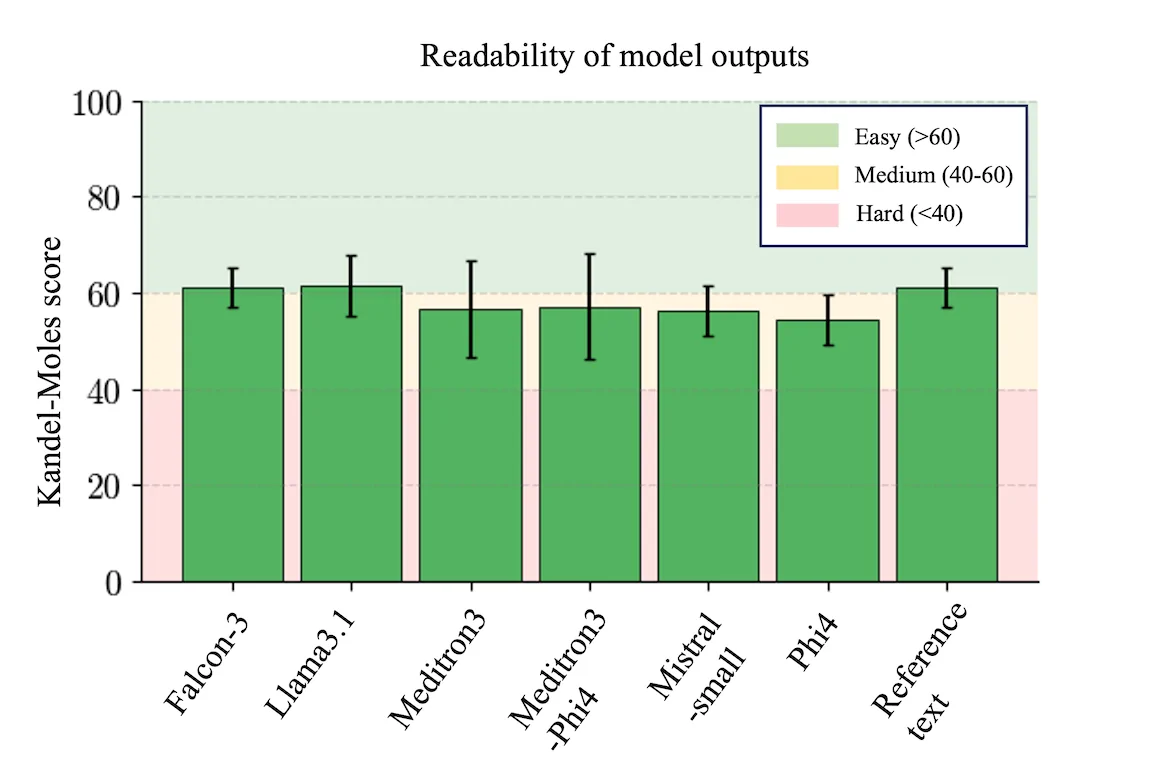
Kandel-Moles readability index of sampled discharge letters (higher values indicate greater readability). Each colored bar represents a tested model. The error bars represent the 95% CI.

To assess the readability of the generated summaries in French, as before, we used the Kandel-Moles readability index. The original discharge letters were found to have a medium readability level (Figure 5). Contrary to expectations, no model achieved a superior readability score (indicated by higher values in Figure 5), and in some instances, the automated generation resulted in text with lower readability than the original.

However, evaluations by clinicians painted a more critical picture (Figure 2). Clinicians rated the generated summaries as “dissatisfied” for readability and “very dissatisfied” for tone. While models performed adequately on the dimensions of diagnostic accuracy, completeness, safety, and privacy (rated “neutral” to “satisfied”), they fell short on treatment plan accuracy, patient instructions, logical structure, and overall report quality (rated below “neutral”). Notably, all models consistently achieved a “very satisfied” rating in the ethics dimension.

We noticed that the Meditron3 family consistently underperformed relative to the other models (Figure 2). This finding is counterintuitive, as these medically fine-tuned models performed substantially worse than the general-purpose base models and Mistral-small. We hypothesize that this performance degradation stems from trade-offs during continual fine-tuning, whereby specialization for medical knowledge may have compromised foundational capabilities such as reasoning, multilingual comprehension, or instruction following. We leave further investigation of this phenomenon to future work.

**Figure 6:**
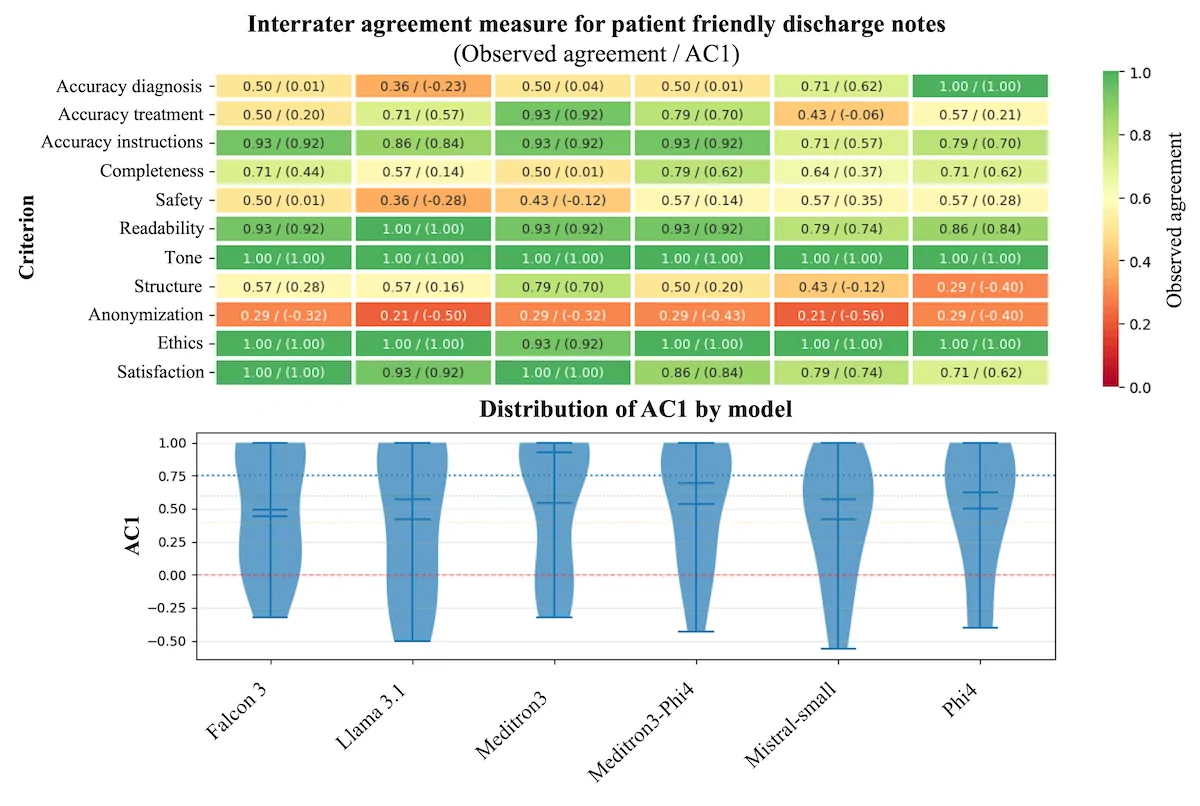
Raters’ agreement for the patient-friendly discharge note generation use case. Interrater agreement was measured by observed agreement and Gwet agreement coefficient, version 1 (AC1). Each column presents the ratings for 1 model; the model name is presented at the bottom of the plot. The color of the heat map represents the observed agreement value. The value in each cell presents both the observed agreement and AC1 value in the format observed agreement/(AC1). The violin plots present the distribution of AC1 values across all evaluation criteria for each language model. Horizontal reference lines indicate standard interpretation thresholds (excellent agreement indicated by the blue dotted line at an AC1 value of 0.75). Wider violins indicate greater variability in AC1 values across evaluation criteria.

As before, interrater agreement was assessed using both observed agreement and Gwet AC1 (Figure 6). Overall, observed agreement was consistently high across most evaluation criteria and models, frequently exceeding 0.70 and reaching perfect agreement (1.00) for dimensions such as tone and ethics. In contrast, AC1 values showed greater variability, with high agreement observed for readability, tone, ethics, and satisfaction (AC1 values 0.73-1.00 across models), indicating strong and consistent rater alignment for these subjective quality dimensions.

For more task-specific criteria, such as accuracy and completeness, agreement was moderate and model dependent, with higher consistency observed for certain models (eg, Meditron3-Phi4 and Phi4) compared with others. Criteria related to safety and anonymization showed low or even negative AC1 values across several models, despite moderate observed agreement. This discrepancy reflects the known sensitivity of chance-corrected metrics to skewed label distributions and suggests that these dimensions were both challenging to assess consistently and prone to imbalance in rating categories.

Interrater agreement was generally higher and more consistent in the clinical decision support use case than in patient-friendly discharge note generation, as reflected by stronger concordance between observed agreement and AC1. In contrast, the patient-oriented use case exhibited greater variability and more frequent discrepancies between the 2 metrics, likely reflecting increased subjectivity and skewed rating distributions in evaluating language quality and communication aspects.

## Discussion

### Principal Findings

Our study evaluated the performance of small, resource-efficient LLMs across multiple clinically relevant tasks, including information extraction, medical text translation, discharge summary generation, and clinical decision support. Overall, although several models demonstrated competence on simple information extraction tasks (eg, needle-in-the-haystack), performance consistently deteriorated as task complexity increased. In particular, tasks requiring nuanced clinical understanding, complex instruction following, or domain-specific reasoning—such as PHI detection, irAE extraction, discharge summarization, and clinical decision support—had low accuracy and poor reliability.

In the PHI detection task, no model demonstrated consistently high performance across all PHI categories, and several models repeatedly failed to identify entities. Qualitative inspection of model outputs suggests that this is a systematic limitation rather than an isolated error. For example, the Meditron3-8B-Instruct model consistently responded with “John Doe” as the detected name for all tested samples. This behavior may be an unintended consequence of safety-oriented posttraining fine-tuning [37] or may reflect inherent constraints of the models for this task. In comparison with the fine-tuned RoBERTa baseline, zero-shot LLMs appear to lack the capacity to reliably detect or generalize across diverse PHI representations. We hypothesize that incorporating a few-shot learning strategy could substantially improve performance.

In the medical text translation use case, our results indicate that while Phi4 excelled in automated metrics, Falcon3, Llama3.1, and Mistral-small consistently received “satisfied” scores in clinician evaluations. Meditron3-Phi4 failed to show efficacy in automated evaluation metrics and produced translations with a high hallucination rate.

None of the evaluated models produced discharge notes that met the satisfaction criteria of either clinicians or patients. Although summaries generally adhered to ethical and safety constraints—likely reflecting safety posttraining—their clinical completeness, correctness, and usefulness were consistently inadequate.

Similarly, performance on clinical decision support tasks was poor across all tested small LLMs. This contrasts sharply with prior reports of strong performance for larger models such as GPT-4 and domain-specialized systems like Med-PaLM 2 on diagnostic benchmarks. Collectively, these results indicate that the evaluated small LLMs are not yet suitable for deployment in clinical applications requiring deep medical understanding, multistep reasoning, or precise instruction adherence.

Taken together, our findings directly address the study’s primary objective: assessing whether small, resource-efficient LLMs are ready for real-world clinical deployment. Despite their theoretical appeal, current models exhibit insufficient performance across most tested use cases, particularly those involving complex clinical cognition.

### Comparison With Prior Work

For clinical documentation, specifically discharge summary generation, our study revealed that the evaluated small LLMs were unable to produce satisfactory summaries for either clinicians or patients. This aligns with the difficulties reported even for larger models. For instance, prior work using a fine-tuned Llama-3 model and commercial GPT-4 and GPT-3.5 models [38,39] identified notable error rates, inaccuracies, and omissions in LLM-generated summaries for clinicians. Our results with small LLMs indicate an even more pronounced performance deficit, suggesting that the intricate task of synthesizing complex medical narratives into accurate and complete clinician-facing summaries likely requires model capacities beyond those of the small LLMs tested.

The contrast is also stark when considering patient-friendly summaries. While studies by Ganzinger et al [38] using GPT-4 and Williams et al [39] with GPT-3.5 demonstrated LLMs’ ability to significantly improve readability and produce largely accurate simplified texts, our small LLMs did not achieve this. This disparity underscores that the architectural scale and training data extent of models like GPT-4 are likely critical for nuanced rephrasing and simplification tasks.

It is possible that the issues we found are less prominent when using large-scale LLMs. Their practical implementation and deployment, however, face significant challenges. Foremost among these are concerns regarding output reliability. For instance, studies deploying even advanced models like GPT-4 for discharge summarization have reported hallucinations or critical detail omissions in approximately 40% of cases [39]. This underscores broader safety and reliability issues, indicating that a substantial proportion of LLM applications in medicine exhibit inaccuracies, rendering them insufficiently reliable for autonomous clinical use without rigorous human oversight. Furthermore, the seamless integration of these models into existing clinical workflows remains largely conceptual, with most current applications being retrospective or prototypical. The “black-box” nature of many LLMs also poses challenges for explainability, a critical factor for trust and adoption in clinical decision-making.

### Limitations

Our study has several limitations that should be considered when interpreting the results and planning for future work. First, to ensure consistency, all experiments were conducted in a zero-shot setting, which may limit the performance of the models. Few-shot prompting may improve performance, especially for information extraction tasks such as NER detection.

Second, our model selection did not include the full and rapidly evolving landscape of small LLMs. Our analysis focused on dense transformer architectures. Consequently, these findings may not generalize to alternative architectures, such as Mixture-of-Experts, or to domain-specialized models (eg, the MedGemma family [40]), which may offer different performance trade-offs.

Third, we observed a systematic failure in the Meditron3-8B-Instruct model in the PHI detection task, characterized by the recurrent generation of placeholders (eg, John Doe) rather than identifying actual entities. We hypothesize that this behavior stems from aggressive safety alignment during posttraining, which may inadvertently interfere with the model’s utility for clinical deidentification tasks. However, as we lacked access to non–safety-tuned checkpoints for a direct comparative analysis, this remains a preliminary observation that requires further investigation. From a practical perspective, these findings highlight the need for mitigation strategies when deploying LLMs for PHI detection. In particular, task-specific supervised fine-tuning on annotated clinical corpora may help recover entity recognition performance. In addition, hybrid approaches that combine rule-based methods (eg, pattern matching or deterministic deidentification pipelines) with model-based extraction could improve robustness and reduce the risk of systematic failures.

Furthermore, the structural heterogeneity of the discharge letters used in the medical translation use case may have influenced the observed performance. Clinician feedback highlighted that some of the sampled letters are from surgical services, which often feature simpler structures and fewer technical sections compared with those from other medical services. Consequently, the inclusion of these relatively simpler documents may have inflated the perceived efficacy of the models. The current evaluation did not stratify results by clinical service or document complexity, which potentially limits the generalizability of our findings to more linguistically dense or multifaceted medical discourse.

Finally, the key limitation of this study is the relatively small sample size used for evaluation. However, the objective was not to perform an exhaustive comparison of models but to assess whether current systems meet a minimum threshold for clinical utility. To mitigate potential sampling bias, cases were randomly selected, and their representativeness was verified using Kolmogorov-Smirnov tests comparing key attributes (eg, visit count, stay duration, and number and length of documents) against the full dataset; no statistically significant differences were observed (all *P*>.16). Details about the test can be found in the supplementary materials. Across these samples, all models exhibited recurrent and clinically significant errors, indicating that they are not yet suitable for deployment in safety-critical settings. From a safety auditing perspective, such failures observed even in a small sample (eg, N=10) are sufficient to demonstrate unreliability. While increasing the sample size would improve the precision of performance estimates, it is unlikely to change the overall qualitative conclusion. Future work will focus on expanding the evaluation cohort through continued data collection with clinician input, as well as systematically quantifying interrater agreement to further assess the robustness and reliability of these inherently subjective evaluations.

### Future Directions

Based on the findings and limitations of our study, we propose several directions for future research.

First, future work could transition from zero-shot evaluations to few-shot prompting, chain-of-thought prompting, and test-time scaling strategies. Such research could investigate how the inclusion of domain-specific examples influences model performance. This could help determine the limits of small LLMs’ capabilities in clinical settings.

A dedicated comparative analysis between base models and their safety-tuned counterparts is necessary to confirm our hypothesis regarding PHI detection failures. This research could focus on the potential loss in clinical task performance due to safety fine-tuning and developing system-level prompts that can bypass unintended refusals while maintaining safety requirements.

Another notable limitation of our methodology is the bilingual disparity between the prompt instructions (English) and the source material (French) for the medical text translation task. Such cross-lingual prompting can introduce confounding effects, as models are often more heavily optimized for instruction following in English than in other languages. To address this, future work should include a comparative analysis of prompt-language effects to determine whether the observed performance deficits are inherent to the translation task or artifacts of the instruction language.

Furthermore, future research should incorporate more granular, stratified samples of clinical notes or letters across diverse clinical specialties. Specifically, there is a need to evaluate model performance on high-complexity letters, such as those from internal medicine or oncology, which often involve complex multimorbidities and nuanced therapeutic reasoning. Future studies should aim to quantify how document length, structural density, and the presence of complex reasoning chains affect performance across medical use cases.

To improve the generalizability of our findings, future work should involve larger, multidisciplinary panels of clinicians from diverse medical specialties. Additionally, conducting patient-side comprehension studies is important to faithfully validate the quality of generated patient-friendly discharge notes.

### Conclusions

The substantial computational resources and stringent data governance requirements associated with large LLMs present considerable barriers for many healthcare institutions. A significant number of hospitals and clinics, particularly those with limited resources or those operating under strict data protection regulations (eg, GDPR and HIPAA), may be unable to deploy large proprietary models locally or use cloud-based APIs due to cost, infrastructure limitations, or data residency and security concerns. It was precisely these practical deployment constraints that motivated our investigation into the clinical utility of smaller, more resource-efficient LLMs.

Our findings demonstrate a critical disconnect between the theoretical promise of smaller LLMs and their practical utility in a specific clinical environment. Although these models offer a feasible deployment pathway for resource-constrained settings, their performance on our local, French-language data was marked by significant failures in complex reasoning and instruction adherence. This discrepancy demonstrates precisely why standardized benchmarks are insufficient; they fail to account for the local specificities and linguistic nuances that dictate real-world efficacy.

The primary contribution of this work is therefore not merely to report on model limitations but to provide health care institutions with a methodological tool, as an evaluation protocol, through which they can generate their own evaluation benchmarks, ensuring that any deployed AI is genuinely fit for its intended clinical purpose. Concretely, this protocol consists of four key steps: (1) task scoping to define use cases and error profiles; (2) local dataset construction using representative, real-world data; (3) multidimensional evaluation of performance, reasoning, and safety; and (4) iterative failure analysis to refine model deployment.

By emphasizing locally grounded validation over generalized performance claims, this protocol enables institutions to move from passive adoption of AI systems to active, evidence-based qualification. In doing so, it establishes a practical pathway toward safer and more context-sensitive integration of language models into clinical workflows.

## Data Availability

The datasets generated or analyzed during this study are not publicly available due to confidentiality and data protection restrictions associated with patient data from Lausanne University Hospital but may be available in de-identified or aggregated form from the corresponding author on reasonable request, subject to institutional approval and applicable data protection regulations.

## Appendix

Multimedia Appendix 1 Full prompt templates and variable definitions used in zero-shot experiments.

Multimedia Appendix 2 Additional experimental results and extended tables.

## Abbreviations

AC1: agreement coefficient, version 1
BF16: Brain Floating Point 16-bit
EU: European Union
FDA: US Food and Drug Administration
FP32: 32-bit floating-point precision
GDPR: General Data Protection Regulation
HIPAA: Health Insurance Portability and Accountability Act
irAE: immune-related adverse event
LLM: large language model
Pen ROUGE: penalized ROUGE
PHI: protected health information
RAG: retrieval-augmented generation
RoBERTa: Robustly Optimized BERT Pretraining Approach
ROUGE: recall-oriented understudy for gisting evaluation
