# Supplementary material for: “Small” Large Language Models in the Hospital: Evaluation Study on Real-World Data in a Resource-Constrained Setting
Source: J Med Internet Res. 2026 Sep 9;28:e86453. doi: 10.2196/86453 (PMC13601877; doi:10.2196/86453)
Supplement: Supplementary file 2 [file jmir_v28i1e86453_app2.docx]

# Multimedia Appendix 2. Additional experimental results and extended tables

## **Statistical Analysis for model performance**

To compare the clinicians’ ratings over models, we performed the Kruskal-Wallis test over the rated model replies for each criterion. We decided to use this test because it is non-parametric and does not assume that the ratings are normally distributed. After conducting the Kruskal-Wallis test, we performed post hoc Pairwise Mann-Whitney U tests between models for each metric. Finally, we performed Bonferroni correction and estimated the effect size using the Cliff’s delta. The detailed test result table for each use case is shown below.

MA-Table 1: Clinical Decision Support

| metric | model_1 | model_2 | median  1 | median  2 | mean  1 | mean  2 | p_value | p_value_adj | significant | cliffsdelta | effect_size |
| --- | --- | --- | --- | --- | --- | --- | --- | --- | --- | --- | --- |
| accuracy treatment | Phi4 | Mistral-small | 3 | 3 | 2.87 | 3.12 | 0.26 | 1 | FALSE | -0.21 | small |
| accuracy treatment | Phi4 | Falcon3 | 3 | 2.5 | 2.87 | 2.43 | 0.17 | 1 | FALSE | 0.26 | small |
| accuracy treatment | Phi4 | Llama3.1 | 3 | 2 | 2.87 | 2.05 | 0.02 | 0.39 | FALSE | 0.43 | medium |
| accuracy treatment | Phi4 | Meditron-3 | 3 | 2 | 2.87 | 1.88 | 0.003 | 0.05 | FALSE | 0.56 | large |
| accuracy treatment | Phi4 | Meditron3-Phi4 | 3 | 2 | 2.87 | 2.12 | 0.01 | 0.16 | FALSE | 0.5 | large |
| accuracy treatment | Mistral-small | Falcon3 | 3 | 2.5 | 3.12 | 2.43 | 0.04 | 0.66 | FALSE | 0.40 | medium |
| accuracy treatment | Mistral-small | Llama3.1 | 3 | 2 | 3.12 | 2.05 | 0.009 | 0.14 | FALSE | 0.51 | large |
| accuracy treatment | Mistral-small | Meditron-3 | 3 | 2 | 3.12 | 1.88 | 0.002 | 0.03 | TRUE | 0.61 | large |
| accuracy treatment | Mistral-small | Meditron3-Phi4 | 3 | 2 | 3.12 | 2.12 | 0.005 | 0.07 | FALSE | 0.56 | large |
| accuracy treatment | Falcon3 | Llama3.1 | 2.5 | 2 | 2.43 | 2.05 | 0.27 | 1 | FALSE | 0.21 | small |
| accuracy treatment | Falcon3 | Meditron-3 | 2.5 | 2 | 2.43 | 1.88 | 0.09 | 1 | FALSE | 0.32 | small |
| accuracy treatment | Falcon3 | Meditron3-Phi4 | 2.5 | 2 | 2.43 | 2.12 | 0.30 | 1 | FALSE | 0.20 | small |
| accuracy treatment | Llama3.1 | Meditron-3 | 2 | 2 | 2.05 | 1.88 | 0.70 | 1 | FALSE | 0.07 | negligible |
| accuracy treatment | Llama3.1 | Meditron3-Phi4 | 2 | 2 | 2.05 | 2.12 | 0.70 | 1 | FALSE | -0.07 | negligible |
| accuracy treatment | Meditron-3 | Meditron3-Phi4 | 2 | 2 | 1.88 | 2.12 | 0.35 | 1 | FALSE | -0.18 | small |
| logical reasoning | Phi4 | Mistral-small | 3 | 3.5 | 3.06 | 3.18 | 0.58 | 1 | FALSE | -0.11 | negligible |
| logical reasoning | Phi4 | Falcon3 | 3 | 2 | 3.06 | 2.64 | 0.34 | 1 | FALSE | 0.19 | small |
| logical reasoning | Phi4 | Llama3.1 | 3 | 3 | 3.06 | 2.23 | 0.03 | 0.56 | FALSE | 0.40 | medium |
| logical reasoning | Phi4 | Meditron-3 | 3 | 2 | 3.06 | 1.93 | 0.003 | 0.05 | FALSE | 0.59 | large |
| logical reasoning | Phi4 | Meditron3-Phi4 | 3 | 2.5 | 3.06 | 2.31 | 0.06 | 1 | FALSE | 0.36 | medium |
| logical reasoning | Mistral-small | Falcon3 | 3.5 | 2 | 3.1875 | 2.64 | 0.23 | 1 | FALSE | 0.23 | small |
| logical reasoning | Mistral-small | Llama3.1 | 3.5 | 3 | 3.18 | 2.23 | 0.01 | 0.24 | FALSE | 0.47 | large |
| logical reasoning | Mistral-small- | Meditron-3 | 3.5 | 2 | 3.18 | 1.93 | 0.005 | 0.08 | FALSE | 0.56 | large |
| logical reasoning | Mistral-small | Meditron3-Phi4 | 3.5 | 2.5 | 3.18 | 2.31 | 0.03 | 0.50 | FALSE | 0.42 | medium |
| logical reasoning | Falcon3 | Llama3.1 | 2 | 3 | 2.64 | 2.23 | 0.36 | 1 | FALSE | 0.17 | small |
| logical reasoning | Falcon3 | Meditron-3 | 2 | 2 | 2.64 | 1.93 | 0.13 | 1 | FALSE | 0.30 | small |
| logical reasoning | Falcon3 | Meditron3-Phi4 | 2 | 2.5 | 2.64 | 2.31 | 0.48 | 1 | FALSE | 0.14 | negligible |
| logical reasoning | Llama3.1 | Meditron-3 | 3 | 2 | 2.23 | 1.93 | 0.36 | 1 | FALSE | 0.18 | small |
| logical reasoning | Llama3.1 | Meditron3-Phi4 | 3 | 2.5 | 2.23 | 2.31 | 0.86 | 1 | FALSE | -0.03 | negligible |
| logical reasoning | Meditron-3 | Meditron3-Phi4 | 2 | 2.5 | 1.93 | 2.31 | 0.30 | 1 | FALSE | -0.21 | small |
| relevancy tests | Phi4 | Mistral-small | 3 | 3 | 2.81 | 2.93 | 0.45 | 1 | FALSE | -0.15 | small |
| relevancy tests | Phi4 | Falcon3 | 3 | 3 | 2.81 | 2.70 | 0.72 | 1 | FALSE | 0.06 | negligible |
| relevancy tests | Phi4 | Llama3.1 | 3 | 2 | 2.81 | 2.23 | 0.08 | 1 | FALSE | 0.34 | medium |
| relevancy tests | Phi4 | Meditron-3 | 3 | 2 | 2.81 | 1.88 | 0.004 | 0.06 | FALSE | 0.55 | large |
| relevancy tests | Phi4 | Meditron3-Phi4 | 3 | 2 | 2.81 | 2.31 | 0.12 | 1 | FALSE | 0.30 | small |
| relevancy tests | Mistral-small | Falcon3 | 3 | 3 | 2.93 | 2.70 | 0.48 | 1 | FALSE | 0.14 | negligible |
| relevancy tests | Mistral-small | Llama3.1 | 3 | 2 | 2.93 | 2.23 | 0.10 | 1 | FALSE | 0.32 | small |
| relevancy tests | Mistral-small | Meditron-3 | 3 | 2 | 2.93 | 1.88 | 0.01 | 0.24 | FALSE | 0.48 | large |
| relevancy tests | Mistral-small | Meditron3-Phi4 | 3 | 2 | 2.93 | 2.31 | 0.14 | 1 | FALSE | 0.3 | small |
| relevancy tests | Falcon3 | Llama3.1-8B | 3 | 2 | 2.70 | 2.23 | 0.24 | 1 | FALSE | 0.23 | small |
| relevancy tests | Falcon3 | Meditron-3 | 3 | 2 | 2.70 | 1.88 | 0.03 | 0.52 | FALSE | 0.41 | medium |
| relevancy tests | Falcon3 | Meditron3-Phi4 | 3 | 2 | 2.70 | 2.31 | 0.33 | 1 | FALSE | 0.19 | small |
| relevancy tests | Llama3.1 | Meditron-3 | 2 | 2 | 2.23 | 1.88 | 0.35 | 1 | FALSE | 0.17 | small |
| relevancy tests | Llama3.1 | Meditron3-Phi4 | 2 | 2 | 2.23 | 2.31 | 0.83 | 1 | FALSE | -0.04 | negligible |
| relevancy tests | Meditron-3 | Meditron3-Phi4 | 2 | 2 | 1.88 | 2.31 | 0.24 | 1 | FALSE | -0.22 | small |
| safety treatment | Phi4 | Mistral-small | 3 | 2.5 | 2.75 | 2.68 | 0.92 | 1 | FALSE | 0.02 | negligible |
| safety treatment | Phi4 | Falcon3 | 3 | 2 | 2.75 | 2.58 | 0.65 | 1 | FALSE | 0.09 | negligible |
| safety treatment | Phi4 | Llama3.1 | 3 | 2 | 2.75 | 2 | 0.03 | 0.52 | FALSE | 0.41 | medium |
| safety treatment | Phi4 | Meditron-3 | 3 | 1 | 2.75 | 1.76 | 0.007 | 0.11 | FALSE | 0.52 | large |
| safety treatment | Phi4 | Meditron3-Phi4 | 3 | 2 | 2.75 | 2.06 | 0.01 | 0.27 | FALSE | 0.46 | medium |
| safety treatment | Mistral-small | Falcon3 | 2.5 | 2 | 2.68 | 2.58 | 0.82 | 1 | FALSE | 0.04 | negligible |
| safety treatment | Mistral-small | Llama3.1 | 2.5 | 2 | 2.68 | 2 | 0.09 | 1 | FALSE | 0.33 | medium |
| safety treatment | Mistral-small | Meditron-3 | 2.5 | 1 | 2.68 | 1.76 | 0.02 | 0.34 | FALSE | 0.44 | medium |
| safety treatment | Mistral-small | Meditron3-Phi4 | 2.5 | 2 | 2.68 | 2.06 | 0.13 | 1 | FALSE | 0.30 | small |
| safety treatment | Falcon3 | Llama3.1 | 2 | 2 | 2.58 | 2 | 0.12 | 1 | FALSE | 0.29 | small |
| safety treatment | Falcon3 | Meditron-3 | 2 | 1 | 2.58 | 1.76 | 0.02 | 0.44 | FALSE | 0.42 | medium |
| safety treatment | Falcon3 | Meditron3-Phi4 | 2 | 2 | 2.58 | 2.06 | 0.15 | 1 | FALSE | 0.27 | small |
| safety treatment | Llama3.1 | Meditron-3 | 2 | 1 | 2 | 1.76 | 0.44 | 1 | FALSE | 0.14 | negligible |
| safety treatment | Llama3.1 | Meditron3-Phi4 | 2 | 2 | 2 | 2.06 | 0.92 | 1 | FALSE | -0.02 | negligible |
| safety treatment | Meditron-3 | Meditron3-Phi4 | 1 | 2 | 1.76 | 2.06 | 0.29 | 1 | FALSE | -0.20 | small |

MA-Table 2: Patient Friendly Discharge Note

| metric | model_1 | model_2 | median_1 | median_2 | mean_1 | mean_2 | p_value | p_value_adj | significant | cliffs_delta | effect_size |
| --- | --- | --- | --- | --- | --- | --- | --- | --- | --- | --- | --- |
| accuracy diagnosis | Phi4 | Mistral-small | 4 | 4 | 4.22 | 3.86 | 0.21 | 1.00 | FALSE | 0.15 | small |
| accuracy diagnosis | Phi4 | Llama3.1 | 4 | 4 | 4.22 | 3.43 | <0.001 | 0.03 | TRUE | 0.42 | medium |
| accuracy diagnosis | Phi4 | Falcon3 | 4 | 4 | 4.22 | 3.14 | <0.001 | 0.06 | FALSE | 0.40 | medium |
| accuracy diagnosis | Phi4 | Meditron3 | 4 | 4 | 4.22 | 3.14 | <0.001 | 0.03 | TRUE | 0.42 | medium |
| accuracy diagnosis | Phi4 | Meditron3-Phi4 | 4 | 4 | 4.22 | 3.04 | <0.001 | <0.001 | TRUE | 0.55 | large |
| accuracy diagnosis | Mistral-small | Llama3.1 | 4 | 4 | 3.86 | 3.43 | 0.08 | 1.00 | FALSE | 0.24 | small |
| accuracy diagnosis | Mistral-small | Falcon3 | 4 | 4 | 3.86 | 3.14 | 0.07 | 1.00 | FALSE | 0.26 | small |
| accuracy diagnosis | Mistral-small | Meditron3 | 4 | 4 | 3.86 | 3.14 | 0.05 | 0.74 | FALSE | 0.27 | small |
| accuracy diagnosis | Mistral-small | Meditron3-Phi4 | 4 | 4 | 3.86 | 3.04 | 0.01 | 0.09 | FALSE | 0.38 | medium |
| accuracy diagnosis | Llama3.1 | Falcon3 | 4 | 4 | 3.43 | 3.14 | 0.50 | 1.00 | FALSE | 0.10 | negligible |
| accuracy diagnosis | Llama3.1 | Meditron3 | 4 | 4 | 3.43 | 3.14 | 0.54 | 1.00 | FALSE | 0.09 | negligible |
| accuracy diagnosis | Llama3.1 | Meditron3-Phi4 | 4 | 4 | 3.43 | 3.04 | 0.25 | 1.00 | FALSE | 0.17 | small |
| accuracy diagnosis | Falcon3 | Meditron3 | 4 | 4 | 3.14 | 3.14 | 0.88 | 1.00 | FALSE | 0.02 | negligible |
| accuracy diagnosis | Falcon3 | Meditron3-Phi4 | 4 | 4 | 3.14 | 3.04 | 0.63 | 1.00 | FALSE | 0.07 | negligible |
| accuracy diagnosis | Meditron3 | Meditron3-Phi4 | 4 | 4 | 3.14 | 3.04 | 0.64 | 1.00 | FALSE | 0.07 | negligible |
| accuracy instructions | Phi4 | Mistral-small | 2 | 2 | 2.18 | 2.36 | 0.55 | 1.00 | FALSE | -0.09 | negligible |
| accuracy instructions | Phi4 | Llama3.1 | 2 | 1 | 2.18 | 1.68 | 0.08 | 1.00 | FALSE | 0.26 | small |
| accuracy instructions | Phi4 | Falcon3 | 2 | 2 | 2.18 | 1.64 | 0.08 | 1.00 | FALSE | 0.25 | small |
| accuracy instructions | Phi4 | Meditron3 | 2 | 1 | 2.18 | 1.26 | <0.001 | <0.001 | TRUE | 0.55 | large |
| accuracy instructions | Phi4 | Meditron3-Phi4 | 2 | 1 | 2.18 | 1.36 | <0.001 | 0.02 | TRUE | 0.46 | medium |
| accuracy instructions | Mistral-small | Llama3.1 | 2 | 1 | 2.36 | 1.68 | 0.03 | 0.38 | FALSE | 0.33 | medium |
| accuracy instructions | Mistral-small | Falcon3 | 2 | 2 | 2.36 | 1.64 | 0.02 | 0.31 | FALSE | 0.34 | medium |
| accuracy instructions | Mistral-small | Meditron3 | 2 | 1 | 2.36 | 1.26 | <0.001 | <0.001 | TRUE | 0.56 | large |
| accuracy instructions | Mistral-small | Meditron3-Phi4 | 2 | 1 | 2.36 | 1.36 | <0.001 | 0.01 | TRUE | 0.49 | large |
| accuracy instructions | Llama3.1 | Falcon3 | 1 | 2 | 1.68 | 1.64 | 0.85 | 1.00 | FALSE | -0.03 | negligible |
| accuracy instructions | Llama3.1 | Meditron3 | 1 | 1 | 1.68 | 1.26 | 0.01 | 0.13 | FALSE | 0.33 | medium |
| accuracy instructions | Llama3.1 | Meditron3-Phi4 | 1 | 1 | 1.68 | 1.36 | 0.10 | 1.00 | FALSE | 0.22 | small |
| accuracy instructions | Falcon3 | Meditron3 | 2 | 1 | 1.64 | 1.26 | <0.001 | 0.04 | TRUE | 0.39 | medium |
| accuracy instructions | Falcon3 | Meditron3-Phi4 | 2 | 1 | 1.64 | 1.36 | 0.05 | 0.74 | FALSE | 0.27 | small |
| accuracy instructions | Meditron3 | Meditron3-Phi4 | 1 | 1 | 1.26 | 1.36 | 0.23 | 1.00 | FALSE | -0.13 | negligible |
| accuracy treatment | Phi4 | Mistral-small | 3 | 3 | 3.00 | 2.89 | 0.82 | 1.00 | FALSE | 0.03 | negligible |
| accuracy treatment | Phi4 | Llama3.1 | 3 | 2 | 3.00 | 2.29 | 0.01 | 0.15 | FALSE | 0.38 | medium |
| accuracy treatment | Phi4 | Falcon3 | 3 | 2 | 3.00 | 2.25 | 0.01 | 0.19 | FALSE | 0.38 | medium |
| accuracy treatment | Phi4 | Meditron3 | 3 | 1 | 3.00 | 1.46 | <0.001 | <0.001 | TRUE | 0.73 | large |
| accuracy treatment | Phi4 | Meditron3-Phi4 | 3 | 2 | 3.00 | 2.14 | 0.01 | 0.09 | FALSE | 0.41 | medium |
| accuracy treatment | Mistral-small | Llama3.1 | 3 | 2 | 2.89 | 2.29 | 0.04 | 0.65 | FALSE | 0.30 | small |
| accuracy treatment | Mistral-small | Falcon3 | 3 | 2 | 2.89 | 2.25 | 0.05 | 0.73 | FALSE | 0.30 | small |
| accuracy treatment | Mistral-small | Meditron3 | 3 | 1 | 2.89 | 1.46 | <0.001 | <0.001 | TRUE | 0.64 | large |
| accuracy treatment | Mistral-small | Meditron3-Phi4 | 3 | 2 | 2.89 | 2.14 | 0.02 | 0.34 | FALSE | 0.35 | medium |
| accuracy treatment | Llama3.1 | Falcon3 | 2 | 2 | 2.29 | 2.25 | 0.74 | 1.00 | FALSE | 0.05 | negligible |
| accuracy treatment | Llama3.1 | Meditron3 | 2 | 1 | 2.29 | 1.46 | <0.001 | 0.01 | TRUE | 0.51 | large |
| accuracy treatment | Llama3.1 | Meditron3-Phi4 | 2 | 2 | 2.29 | 2.14 | 0.48 | 1.00 | FALSE | 0.10 | negligible |
| accuracy treatment | Falcon3 | Meditron3 | 2 | 1 | 2.25 | 1.46 | <0.001 | 0.04 | TRUE | 0.43 | medium |
| accuracy treatment | Falcon3 | Meditron3-Phi4 | 2 | 2 | 2.25 | 2.14 | 0.67 | 1.00 | FALSE | 0.06 | negligible |
| accuracy treatment | Meditron3 | Meditron3-Phi4 | 1 | 2 | 1.46 | 2.14 | 0.01 | 0.20 | FALSE | -0.35 | medium |
| readability | Phi4 | Mistral-small | 2 | 2 | 1.86 | 1.96 | 0.78 | 1.00 | FALSE | -0.04 | negligible |
| readability | Phi4 | Llama3.1 | 2 | 2 | 1.86 | 1.59 | 0.28 | 1.00 | FALSE | 0.15 | small |
| readability | Phi4 | Falcon3 | 2 | 1 | 1.86 | 1.57 | 0.12 | 1.00 | FALSE | 0.22 | small |
| readability | Phi4 | Meditron3 | 2 | 1 | 1.86 | 1.39 | <0.001 | 0.05 | FALSE | 0.41 | medium |
| readability | Phi4 | Meditron3-Phi4 | 2 | 1 | 1.86 | 1.33 | <0.001 | 0.04 | TRUE | 0.42 | medium |
| readability | Mistral-small | Llama3.1 | 2 | 2 | 1.96 | 1.59 | 0.20 | 1.00 | FALSE | 0.18 | small |
| readability | Mistral-small | Falcon3 | 2 | 1 | 1.96 | 1.57 | 0.09 | 1.00 | FALSE | 0.24 | small |
| readability | Mistral-small | Meditron3 | 2 | 1 | 1.96 | 1.39 | <0.001 | 0.05 | FALSE | 0.41 | medium |
| readability | Mistral-small | Meditron3-Phi4 | 2 | 1 | 1.96 | 1.33 | <0.001 | 0.04 | TRUE | 0.43 | medium |
| readability | Llama3.1 | Falcon3 | 2 | 1 | 1.59 | 1.57 | 0.54 | 1.00 | FALSE | 0.09 | negligible |
| readability | Llama3.1 | Meditron3 | 2 | 1 | 1.59 | 1.39 | 0.03 | 0.42 | FALSE | 0.30 | small |
| readability | Llama3.1 | Meditron3-Phi4 | 2 | 1 | 1.59 | 1.33 | 0.02 | 0.35 | FALSE | 0.31 | small |
| readability | Falcon3 | Meditron3 | 1 | 1 | 1.57 | 1.39 | 0.13 | 1.00 | FALSE | 0.20 | small |
| readability | Falcon3 | Meditron3-Phi4 | 1 | 1 | 1.57 | 1.33 | 0.12 | 1.00 | FALSE | 0.21 | small |
| readability | Meditron3 | Meditron3-Phi4 | 1 | 1 | 1.39 | 1.33 | 1.00 | 1.00 | FALSE | 0.00 | negligible |
| satisfaction | Phi4 | Mistral-small | 2 | 2 | 2.14 | 2.11 | 0.95 | 1.00 | FALSE | 0.01 | negligible |
| satisfaction | Phi4 | Llama3.1 | 2 | 1 | 2.14 | 1.57 | 0.02 | 0.33 | FALSE | 0.33 | medium |
| satisfaction | Phi4 | Falcon3 | 2 | 1 | 2.14 | 1.50 | 0.01 | 0.18 | FALSE | 0.36 | medium |
| satisfaction | Phi4 | Meditron3 | 2 | 1 | 2.14 | 1.32 | <0.001 | <0.001 | TRUE | 0.53 | large |
| satisfaction | Phi4 | Meditron3-Phi4 | 2 | 1 | 2.14 | 1.56 | 0.01 | 0.21 | FALSE | 0.36 | medium |
| satisfaction | Mistral-small | Llama3.1 | 2 | 1 | 2.11 | 1.57 | 0.02 | 0.35 | FALSE | 0.33 | small |
| satisfaction | Mistral-small | Falcon3 | 2 | 1 | 2.11 | 1.50 | 0.01 | 0.18 | FALSE | 0.36 | medium |
| satisfaction | Mistral-small | Meditron3 | 2 | 1 | 2.11 | 1.32 | <0.001 | <0.001 | TRUE | 0.52 | large |
| satisfaction | Mistral-small | Meditron3-Phi4 | 2 | 1 | 2.11 | 1.56 | 0.01 | 0.22 | FALSE | 0.36 | medium |
| satisfaction | Llama3.1 | Falcon3 | 1 | 1 | 1.57 | 1.50 | 0.90 | 1.00 | FALSE | 0.02 | negligible |
| satisfaction | Llama3.1 | Meditron3 | 1 | 1 | 1.57 | 1.32 | 0.04 | 0.61 | FALSE | 0.26 | small |
| satisfaction | Llama3.1 | Meditron3-Phi4 | 1 | 1 | 1.57 | 1.56 | 0.73 | 1.00 | FALSE | 0.05 | negligible |
| satisfaction | Falcon3 | Meditron3 | 1 | 1 | 1.50 | 1.32 | 0.05 | 0.68 | FALSE | 0.26 | small |
| satisfaction | Falcon3 | Meditron3-Phi4 | 1 | 1 | 1.50 | 1.56 | 0.81 | 1.00 | FALSE | 0.03 | negligible |
| satisfaction | Meditron3 | Meditron3-Phi4 | 1 | 1 | 1.32 | 1.56 | 0.09 | 1.00 | FALSE | -0.21 | small |
| structure | Phi4 | Mistral-small | 3 | 2.5 | 3.18 | 2.96 | 0.51 | 1.00 | FALSE | 0.10 | negligible |
| structure | Phi4 | Llama3.1 | 3 | 3 | 3.18 | 2.82 | 0.32 | 1.00 | FALSE | 0.15 | small |
| structure | Phi4 | Falcon3 | 3 | 2 | 3.18 | 2.54 | 0.06 | 0.92 | FALSE | 0.28 | small |
| structure | Phi4 | Meditron3 | 3 | 1 | 3.18 | 2.00 | <0.001 | 0.01 | TRUE | 0.50 | large |
| structure | Phi4 | Meditron3-Phi4 | 3 | 2 | 3.18 | 2.56 | 0.07 | 1.00 | FALSE | 0.27 | small |
| structure | Mistral-small | Llama3.1 | 2.5 | 3 | 2.96 | 2.82 | 0.67 | 1.00 | FALSE | 0.06 | negligible |
| structure | Mistral-small | Falcon3 | 2.5 | 2 | 2.96 | 2.54 | 0.19 | 1.00 | FALSE | 0.19 | small |
| structure | Mistral-small | Meditron3 | 2.5 | 1 | 2.96 | 2.00 | <0.001 | 0.04 | TRUE | 0.46 | medium |
| structure | Mistral-small | Meditron3-Phi4 | 2.5 | 2 | 2.96 | 2.56 | 0.23 | 1.00 | FALSE | 0.18 | small |
| structure | Llama3.1 | Falcon3 | 3 | 2 | 2.82 | 2.54 | 0.36 | 1.00 | FALSE | 0.14 | negligible |
| structure | Llama3.1 | Meditron3 | 3 | 1 | 2.82 | 2.00 | 0.01 | 0.18 | FALSE | 0.38 | medium |
| structure | Llama3.1 | Meditron3-Phi4 | 3 | 2 | 2.82 | 2.56 | 0.38 | 1.00 | FALSE | 0.13 | negligible |
| structure | Falcon3 | Meditron3 | 2 | 1 | 2.54 | 2.00 | 0.04 | 0.54 | FALSE | 0.31 | small |
| structure | Falcon3 | Meditron3-Phi4 | 2 | 2 | 2.54 | 2.56 | 0.92 | 1.00 | FALSE | -0.02 | negligible |
| structure | Meditron3 | Meditron3-Phi4 | 1 | 2 | 2.00 | 2.56 | 0.03 | 0.41 | FALSE | -0.33 | medium |

## **Validation on the sample size using Kolmogorov-Smirnov (KS) test**

To assess the representativeness of the randomly selected cases (N=10 and N=14), we performed a Kolmogorov-Smirnov (KS) test comparing the sampled subsets to the full dataset population. We conducted the comparison across key attributes, including the average number of documents per patient, average document length, visit count, and length of stay.

The results of the KS tests (MA-Table 3) did not indicate statistically significant differences between the sampled subsets and the overall dataset for these attributes, suggesting that the random sampling procedure did not introduce systematic bias toward structurally simpler or more complex cases.

The selected sample sizes were intended to support an initial evaluation of model behavior across diverse clinical scenarios. In this context, the analysis focuses on identifying the presence of clinically relevant errors and performance limitations, rather than estimating precise population-level performance metrics.

MA-Table 3: Kolmogorov-Smirnov (KS) test results

|  | Clinical Decision Support (N=10) | Patient Summary (N=14) |
| --- | --- | --- |
| Visit count | KS Statistic: 0.3380  P-value: 0.2120 | KS Statistic: 0.3282  P-value: 0.3609 |
| Stay duration | KS Statistic: 0.2981  P-value: 0.3433 | KS Statistic: 0.3403  P-value: 0.3191 |
| Average number of documents | KS Statistic: 0.3513  P-value: 0.1781 | KS Statistic: 0.2750  P-value: 0.5752 |
| Average document length | KS Statistic: 0.3568  P-value: 0.1652 | KS Statistic: 0.3599  P-value: 0.2585 |

Although the evaluation was conducted on a limited number of randomly sampled cases, the consistent observation of clinically relevant errors across models provides strong evidence of their current limitations in safety-critical settings. In the context of clinical decision support, even a small number of such failures raises concerns about reliability and suitability for deployment. While larger sample sizes would allow for more precise estimation of performance metrics, they are unlikely to alter the overall conclusion that current models do not yet meet the requirements for safe use in hospital environments.
